# Differential Diagnosis of Parotid Lipoma in a Breast Ca Patient

**DOI:** 10.1155/2017/9741828

**Published:** 2017-01-31

**Authors:** Melda Misirlioglu, Yagmur Yilmaz Akyil, Mehmet Zahit Adisen, Alime Okkesim

**Affiliations:** Department of Oral and Maxillofacial Radiology, Faculty of Dentistry, Kırıkkale University, Kırıkkale, Turkey

## Abstract

Lipomas are common benign tumors usually detected on the torso, neck, upper thighs, and upper arms. However, they are rarely found in the parotid gland region. Because of their rarity at this site, they are not often considered in the differential diagnosis of parotid tumors. This report describes a rare case of a lipoma in the superficial lobe of parotid gland. A 71-year-old female patient admitted to our department complaining about swelling and pain in the posterior area of the left mandibular region since one month. Her medical history included mastectomy after breast CA fifteen years ago. Clinical examination revealed a smooth-surfaced, soft, and painful mass, with well-defined margins in the left mandibular region. Differential diagnosis of metastasis, inflammatory neck swellings, and benign salivary gland tumors were considered for the patient. Advanced imaging methods such as ultrasonography and contrast tomography revealed that the lesion was a lipoma of parotid gland. A surgical intervention under general anesthesia was planned for the removal of the mass; however patient refused the surgical treatment. Patient was placed on six-month periodic recall. This article reviews the radiographic appearance and differential diagnoses of lipoma in this rare location.

## 1. Introduction

The ordinary lipomas are the most common neoplasms of mesenchymal origin [1, 2]. They result due to proliferation of normal adipose tissue. Only 15% of lipomas are found in the head and neck region and they usually occur subcutaneously in the posterior neck [1]. Less commonly they can be found in the anterior neck, infratemporal fossa, submandibular space, pharynx, larynx, and parotid gland and in or around the oral cavity [2, 3]. The incidence of lipoma among parotid tumors ranges from 0.6% to 4.4%, with most series reporting an incidence of 1% [4]. The most common origin of these tumors, in the parotid gland, can be single or multiple and is rarely observed in the deep lobe less than superficial lobe. Lipomas are asymptomatic tumors. However if they grow to a large size, they can interfere with mastication and speaking [5]. Lipomas of parotid generally occur in the sixth decade. Advanced imaging methods such as ultrasonography (US), magnetic resonance imaging (MRI), and computed tomography (CT) are used for diagnosis of lipomas [1, 6]. This report describes differential diagnosis of a parotid lipoma in a breast CA patient detected with advanced imaging methods such as US and contrast tomography.

## 2. Case Report

**Figure 1:**
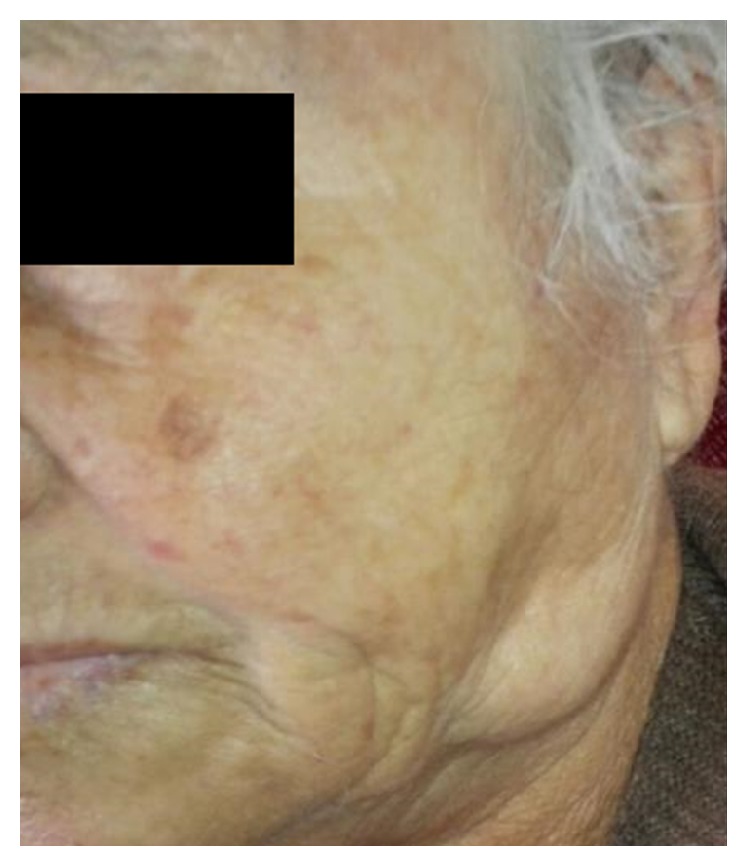
Patient extra-oral photograph showed a smooth-surfaced, soft, and painful mass, with well-defined margins in the right mandibular region.

**Figure 2:**
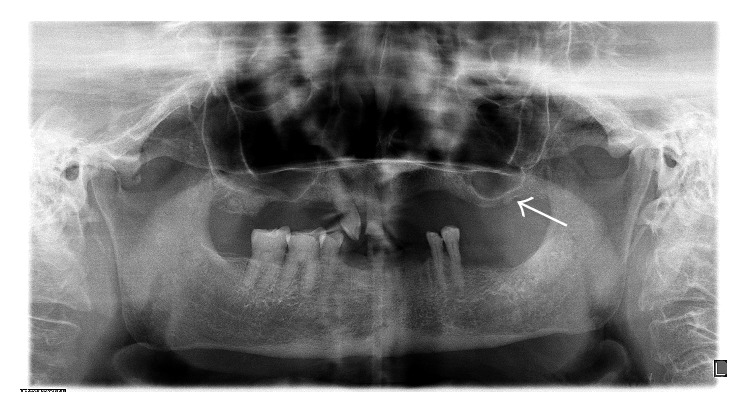
Panoramic radiograph of this patient; root remnant (white arrow) was detected in the left maxillary molar area.

**Figure 3:**
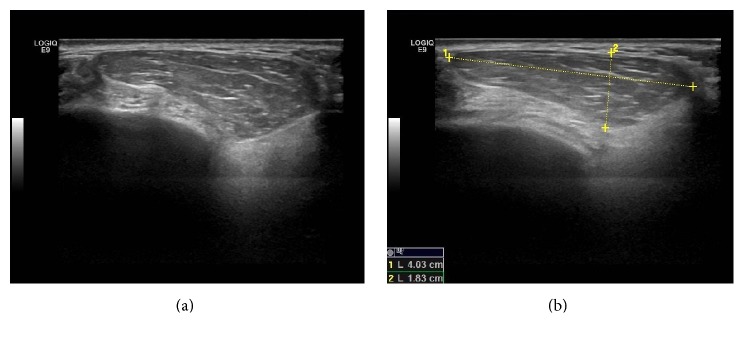
(a) Ultrasound image of the patient showing a well-defined, hypoechoic solid lesion in her superficial lobe of the parotid gland. The lesion had echogenic septas and acoustic empowerment over the posterior region. (b) The lesion was measured approximately 40 × 18 mm in size.

**Figure 4:**
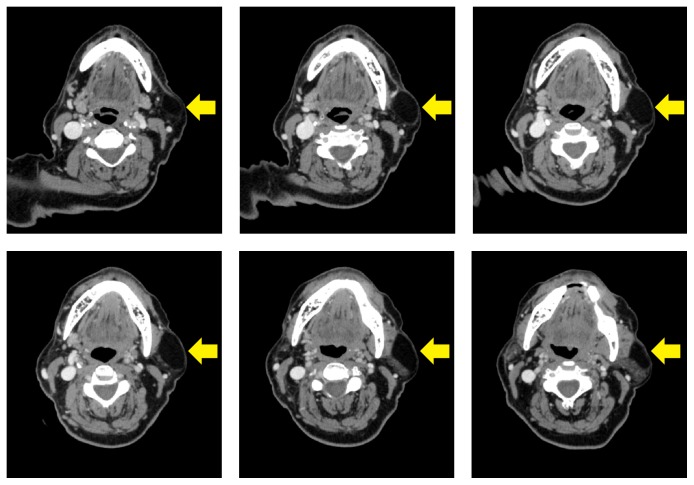
CT scans showed a low density homogeneous capsulated mass with sharp margins in the superficial lobe of the left parotid gland.

A 71-year-old woman patient presented to the Department of Oral and Maxillofacial Radiology with a primary complaint of swelling and pain in the posterior area of the left mandibular region since one month. Patient history revealed that the swelling had been slowly increasing in size. The patient had pain at left side of her face but she cannot distinguish the exact localization. Her medical history included mastectomy after breast CA fifteen years ago. She also has diabetes and is using insulin. Clinical examination revealed a smooth-surfaced, soft, and painful mass, with well-defined margins in the left mandibular region (Figure 1). The swelling was not fixed to the skin and the underlying bone. In her panoramic radiographic examination, root remnant was detected in the left maxillary molar area, possibly related to pain in her face (Figure 2). After taking informed consent of the patient, she was referred to US for differential diagnosis of soft tissue pathologies including metastasis, inflammatory neck swellings, and benign salivary gland tumors. US of the neck region showed bilateral submandibular and parotid glands were normal in size with homogen ecogenity. Thyroid gland was normal with normal ecogenity. However, there was a 40 × 18 mm size, well-defined, hypoechoic solid lesion in her superficial lobe of the parotid gland. The lesion had echogenic septas and acoustic empowerment over the posterior region (Figure 3). Hence a pleomorphic adenoma was suspected and contrast CT was requested. In contrast to CT images, lesion was diagnosed as lipoma due to well-demarcated, hypodense density (Figure 4). A surgical intervention under general anesthesia was planned for the removal of the mass; however patient refused the surgical treatment. Hence, only root remnant was extracted under local anesthesia. Follow-up examination was uneventful and pain was regressed. Patient was placed on a periodic recall.

## 3. Discussion

Lipoma of salivary glands is quite rare with the highest frequency reported in parotid gland that presents normally adipose tissue. Heredity, obesity, diabetes, trauma, radiation, endocrine disorder, insulin injection, and corticosteroid therapy are occasionally implicated as a possible etiologic factors of lipoma [3, 6].

Diagnostic imaging techniques such as US, MRI, and CT help to differentiate lipomas from other soft tissue lesions while identifying the nature and exact location of lesion. For the masses in the salivary glands area, sialography, US, and radionuclide scanning are all of value [7]. US can give a clear and fast diagnosis of lipoma [8]. It can be used as the initial study and shows a homogenous lesion that can be ovoid or lobulated [1]. Lipomas are hypoechoic relative to the adjacent muscle and contain linear echoic lines with no distal enhancement or attenuation. In most cases, they have a clearly identified capsule [8]. In order to determine whether the mass has a glandular origin, the radionuclide scan or the sialogram are usually performed. These imaging techniques can localize the mass inside or outside the salivary gland [7, 9]. Moreover, the radionuclide scan can identify the functional activity of the mass [7]. CT of the neck, which is a helpful imaging method, may differentiate solid masses from cystic masses. It can also be performed for identification of free nodal lesions, localization of the masses within salivary glands, and differentiation of congenital vascular lesions from the lymph nodal chain [1, 7]. Contrast-enhanced high resolution CT is another useful radiological technique in differential diagnosis [10]. While a positive density is observed in normal parotid tissue, a well-demarcated hypodense density (−50 to −150 Hounsfield units) can be identified in lipomatous tissue in contrast-enhanced images [1, 10]. In MRI examinations, lipomas show a similar signal intensity with subcutaneous fat, characterized by a high T1 and low T2 signal intensity [10]. Lipomatous lesions can be clearly distinguished from other types of tumors with the fat suppression sequence of MRI, which provides superior soft tissue definition. It can also reveal the accurate relationship of tumor with facial nerve [10].

The principle consideration in the differential diagnosis of a mass in the parotid region is whether the salivary gland neoplasia is benign or malign. The primary differential diagnosis of neck masses as benign lesions in the subcutaneous location is a sebaceous cyst or an abscess. Sebaceous cysts are also rounded and subcutaneous. Abscesses typically have overlying induration and erythema [11]. Other benign connective tissue lesions in differential diagnosis include granular cell tumor, traumatic fibroma, neurofibroma, and salivary gland lesions (mucocele and mixed tumor) [5]. Lymphadenopathy is also a common finding in neck area, caused by bacterial or viral infections of the upper respiratory tract. Moreover, cervical tularemia, tuberculosis, brucellosis, or cat scratch disease has to be considered in differential diagnosis of neck masses. Granulomatous inflammatory disease usually occurs in specific age groups and locations. So, the physician should keep this in mind when evaluating a neck mass in clinical examination [7, 12, 13]. Sialolipoma is a new variant of salivary gland lipoma, consisting of both adipose and glandular tissues. Lipoma and sialolipoma can be differentiated from one another microscopically by the lack of entrapment of normal salivary gland acini and ducts [13, 14].

Unless proven otherwise, any unknown neck mass, particularly symptom-free, located unilaterally and related with a known lymph node groups, must be evaluated as a metastatic lesion [7]. Liposarcoma, malignant counterpart of lipoma, is especially important to consider in differential diagnosis [15]. Nevertheless, it is rarely found in this region. MRI can accurately distinguish between lipomas and liposarcomas [9]. While lipoma shows a homogeneous appearance in MRI images, liposarcoma appears more heterogeneous and is enhanced following injection of contrast medium [1]. Hence MRI with contrast enhancement can be performed to rule out the possibility of liposarcoma, when the patient is decided to be followed up.

Lipomas usually are not treated, because most of them are asymptomatic. Only for esthetic reasons or complaints like paresthesia, lipoma has to be removed surgically [16]. In this case, patient claimed to have pain in lesion area; however the pain was relieved after extraction of inflamed root fragments. Also diabetes may be effective as a cause of pain in this case. Differential diagnosis of neck swellings become very important in suspicious cases. The spread of head and neck carcinoma is similar to inflammatory disease, generally following an orderly lymphatic spread. Metastasis lymph node is also seen similar to this neck swelling [7]. In this case our patient's medical history included breast CA and neck swelling was suspicious about the metastasis. However advanced imaging methods revealed the presence of lipoma in parotid gland. This case emphasizes the need for the oral health care professionals to be familiar with the clinical manifestations and radiological findings of neck swellings and differential diagnosis of lipomas with other benign and malignant lesions.
